# Prevalence and outcome of HIV infected children admitted in a tertiary hospital in Northern Tanzania

**DOI:** 10.1186/s12887-022-03105-8

**Published:** 2022-02-21

**Authors:** Tulla S. Masoza, Raphael Rwezaula, Delfina R. Msanga, Neema Chami, Julieth Kabirigi, Emmanuela Ambrose, Restituta Muro, Stella Mongella, Adolfine Hokororo, Elizabeth Kwiyolecha, Robert Peck

**Affiliations:** 1 Department of Pediatrics and Child Health, Catholic University of Health and Allied Sciences –Bugando, P.O Box 1464, Mwanza, Tanzania; 2 Department of Pediatrics and Child Health, Bugando Medical Centre, P.O. Box 1370, Mwanza, Tanzania; 3 Jakaya Kikwete Cardiac Institute, P.O Box 65141, Dar-es Salaam, Tanzania

**Keywords:** HIV infection, HIV infected children, PITC, Outcome

## Abstract

**Background:**

Provider Initiated Testing and Counseling (PITC) among hospitalized children have shown to increase the probability of identifying HIV-infected children and hence be able to link them to HIV care. We aimed at determining the prevalence, clinical characteristics and outcome of HIV-infected children admitted at Bugando Medical Centre (BMC) after active provision of PITC services.

**Methods:**

A cross-sectional study with follow up at three months post enrollment was done. Children with unknown HIV status were tested for HIV infection as per 2012 Tanzanian algorithm. Questionnaires were used to collect demographic, clinical and follow up information. Data was statistically analyzed in STATA v13.

**Results:**

A total of 525 children were enrolled in the study. Median [IQR] age was 28 [15–54] months. Males consisted of 60.2% of all the participants. HIV prevalence was 9.3% (49/525). Thirty-three (67.3%) of HIV-infected children were newly diagnosed at enrolment. Thirty-nine (79.6%) of all HIV-infected patients had WHO HIV/AIDS clinical stage four disease, 10 (20.4%) had WHO clinical stage three and none qualified in stage one or two. About 84% (41/49) of HIV infected children had severe immunodeficiency at the time of the study. Factors that were independently associated with HIV infection were, cough (OR 2.40 [1.08–5.31], *p* = 0.031), oral thrush (OR 20.06[8.29–48.52], *p* < 0.001), generalized lymphadenopathy (OR 5.61 [1.06–29.56], *p* = 0.042), severe acute malnutrition (OR 6.78 [2.28–20.12], *p* = 0.001), severe stunting (OR 9.09[2.80–29.53], *p* = 0.034) and death of one or both parents (OR 3.62 [1.10–11.87], *p* = 0.034). The overall mortality (in-hospital and post-hospital) was 38.8% among HIV-infected children compared with 14.0% in HIV-uninfected children. Within three months period after discharge from the hospital, 71.4% (25/35) of discharged HIV-infected children reported to have attended HIV clinic at least once and 60.0% (21/35) were on antiretroviral medications.

**Conclusion:**

PITC to all admitted children identified significant number of HIV-infected children. Mortality among HIV-infected children is high compared to HIV-uninfected. At the time of follow up about 30% of discharged HIV-infected children did not attend to any HIV care and treatment clinics. Therefore effective efforts are needed to guarantee early diagnosis and linkage to HIV care so as to reduce morbidity and mortality among these children.

## Background

Globally an estimated 3.3 million children below 15 years of age were living with HIV in 2012, among them 260,000 children were newly infected. More than 90% of children infected with HIV are homed in sub-Saharan Africa (SSA) [1] and very few of HIV-exposed infants are identified and about 15% of them are timely tested for HIV infection [2]. In Tanzania by 2006, there were 1.4 million people living with HIV, and approximately 11% were children under 15 years of age [3]. Tanzania HIV/AIDS and Malaria Indicator Survey of 2011-12, reported a 5.1% national prevalence of HIV infection among adults aged 15 to 49 years of age, with prevalence of 4.2% in Mwanza region. HIV prevalence among children aged below 15 years was not reported [4]. However the prevalence of HIV infection among hospitalized children have ranged from 19% to 12% as documented in studies done within few regions of Tanzania from 1996 to 2012 [5–7]. Although the number of new infections globally has dropped by 52% from 2001 to 2012, there is still much effort to be made to realize the dream of a HIV free generation [1], particularly in Tanzania and SSA. Paediatric HIV infection is predominantly acquired through vertical transmission, at time of delivery and through breastfeeding. HIV-infected children are at a higher risk of being hospitalized due to common childhood illnesses like pneumonia and malnutrition with associated high mortality compared to their uninfected counterparts, and majority of the deaths occurring before their second birthday if not initiated on antiretroviral therapy [8, 9]. In order to enhance the survival of HIV-infected children, early diagnosis is important as it is a crucial step towards linkage to HIV care and initiation of treatment with ARV and other prophylactic drugs such as co-trimoxazole and isoniazid. Due to poor linkages and follow up patients present late with advanced HIV disease. A study done among adults in the Lake zone of Tanzania, reported a 59.3% of late diagnosis of HIV infection with 78.7% of them being in stage 3 and 4 of the disease who were likely to be diagnosed following PITC services [10].Linkage to care is essential to HIV-infected individuals, because it ensures that they benefit from the referral and appropriate follow up services available at HIV care and treatment centres. Examples of such services include receiving prophylactic co-trimoxazole therapy and/or ART, screening, prevention and management HIV related co-infections and co-morbidities. By the end of the year 2012 in Mwanza region-Tanzania, the cumulative number of children who were enrolled into HIV care and treatment was 8104 and of these, 4348 (53.6%) were receiving Antiretroviral therapy and a total of 115,620 adults were enrolled into HIV care and of these, 66,425 (57.5%) were on Antiretroviral therapy [11].

Pediatric HIV is understudied at Bugando Medical Centre (BMC) and the actual burden of HIV infection among admitted children is not known. At the time of the study, less than 30% of admitted children with unknown HIV status were receiving PITC services meaning probably there was a significant number of children who were being admitted and possibly readmitted with undiagnosed HIV infection. At BMC, when a child is newly diagnosed with HIV infection or exposure during admission the attending team consult health care providers at Baylor College of Medicine Children's Foundation- Mwanza which is a center affiliated with BMC that offers paediatric HIV care, for review and further plans. Upon their discharge, caretakers are either instructed to attend either Baylor College of Medicine Children's Foundation-Mwanza or at a nearby HIV Care and Treatment Centre (CTC) for follow up. However, it is not known yet of their post hospital discharge progress and whether they do attend and adhere to the follow up visits at HIV care and treatment clinics.

Therefore this study was done to understand the burden of HIV infection among admitted children at Bugando Medical Centre after active PITC; in-hospital andpost- hospital outcome of admitted HIV infected children such mortality, attendance to HIV care and treatment clinics and initiation of antiretroviral therapy after discharge.

## Methods

This was a hospital-based cross-sectional study with follow up at 3 months post enrollment done among children aged one month to twelve years admitted in the department of Paediatrics and Child Health at BMC from August 2014 to February 2015. BMC is a University teaching and a tertiary referral hospital situated along the shores of Lake Victoria in Mwanza City. It is a referral centre for tertiary specialist care for eight regions in Lake and Western zones of Tanzania, including Mwanza, Geita, Simiyu, Mara, Kagera, Shinyanga, Tabora and Kigoma, serving a catchment population of approximately 16 million people. The hospital works in close partnership with Baylor College of Medicine Children's Foundation-Mwanza which offers outpatient paediatric HIV care and treatment services. There are several other HIV care and treatment centers in Mwanza region located in the regional and district hospitals offering both paediatric and adult care.

### Enrolment procedures

For all children whose parents/guardians consented, a structured data collection form was used to obtain demographic information, medical history of participants, physical examination findings and investigation results. A rapid antibody test for HIV was performed to every child with a previous negative or unknown HIV status. HIV testing was conducted according to the standard diagnostic HIV serological testing algorithm of 2012 as recommended by the Tanzanian Ministry of Health [12]. Determine HIV1/2 (Alere Medical Co. Ltd Japan) was used as the first antibody test and Unigold (Trinity Biotech PLC, Bray, Ireland) was used as the second antibody test. Rapid antibody tests were used to identify HIV exposed children (children below 18 months of age) and to confirm HIV infection in children aged above 18 months. A dried blood spot from identified HIV exposed children was obtained for HIV DNA PCR that was performed at BMC laboratory. For HIV seropositive children, a venous blood sample for CD_4_ count or CD_4_ percent in children below five years was collected. Pre and post-test counseling was done by trained nurses and doctors present in the pediatric wards.

### Follow up period

At the time of the enrollment three different phone numbers were recorded from each participant; the participant’s parent/caregiver (if any), the next of kin’s and neighbor’s phone number. Three months after discharge from the hospital, children were traced by phone call and important information about their general status and clinic visits were enquired from their parents/caretaker. Additionally for HIV-infected children information on visits to Care and Treatment Clinic, initiation of ART and/or Cotrimoxazole were enquired and documented. Parents/caretakers were asked to counter-check this information from the clinic cards in order to reduce recall bias. In order to minimize lost to follow up, when the participant was not reachable at the end of the three months period, she/he was traced daily through the phone numbers provided for a period of two weeks. If the participant was not reachable after this period he/she was declared loss to follow up.

### Data analysis

Data was entered into Microsoft Excel and analyzed using STATA version 13. Results were summarized using proportions (%) for categorical data and medians [IQR] for continuous variables. Categorical variables were compared using Chi–square and continuous variables were compared using either the students t-test or the rank sum test depending on the distribution of the variables, where *p*-value of less than 0.05 was considered statistically significant. Multivariate logistic regression analysis was performed to determine features associated with HIV-infection after considering prior knowledge of confounders and results of univariate logistic regression analysis. Odds ratios with respective 95% confidence interval (CI) were reported and *p*-value of less than 0.05 was considered statistically significant.

## Results

### Participants’ enrollment and demographic characteristics

**Fig. 1:**
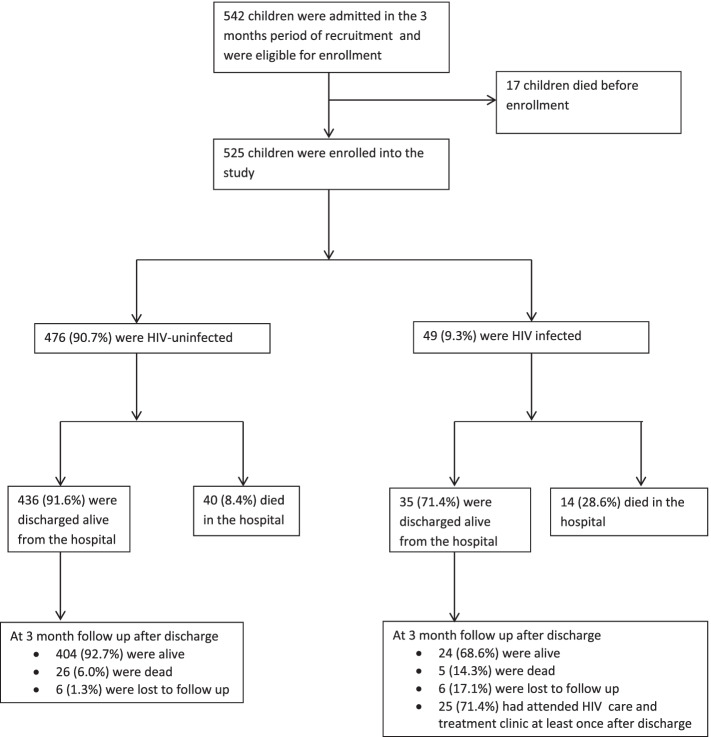
A flow diagram of the participants’ enrolment and outcome

Five hundred and forty two admitted children were eligible for recruitment out of which 17 (3.1%) children were excluded due to death before enrollment. Therefore 525 (96.9%) children were included in the final analysis. None of the participants were excluded due to lack of consent to participate in the study (Fig. 1). There was a male preponderance of 316 (60.2%) among the study participants with median age of 28 [IQR 15-56] and majority of them were residing in Mwanza city. (Table 1).

**Table 1:** Demographic and other baseline characteristics of 525 children aged 1 month to 12 years

Characteristics	HIV-infected Number: (%): or Median [IQR] *N* = 49	HIV-uninfected Number: (%): or Median [IQR] *N* = 476	*P*- Value
**Male gender**	30 (61.2%)	286 (60.1%)	0.877
**Age** (in months)	23 [9–27]	29 [15–60]	<0.001
**Residence**			
Mwanza region	41 (83.7%)	363 (76.3%)	0.241
Out of Mwanza region	8(16.3%)	113 (23.7%)	
**Caregiver's level of education**			
≤ 7 years in school	7 (14.3%)	82 (17.2%)	0.601
> 7 years in school	42 (85.7%)	394 (82.8%)	
**Vital status of parents**			
Parents alive	40 (81.6%)	447 (93.9%)	0.002
One or both parents deceased	9 (18.4%)	29 (6.1%)	
**Mother's HIV status during pregnancy or delivery**			
HIV negative	8 (16.3%)	307 (64.5%)	< 0.001
HIV positive	14 (28.6%)	16 (3.4%)	
Unknown HIV status	27 (55.1%)	153 (32.1%)	
**HIV-infected mothers who received HAART during pregnancy**	6/14 (42.9%)	14/16 (87.5%)	0.01
**Breastfeeding status**			
Never breastfed	1 (2%)	5 (1.1%)	0.609
Still breastfeeding	16 (32.7%)	124 (26.0%)	
Stopped breastfeeding < 3 months ago	5 (10.2%)	41 (8.6%)	
Stopped breastfeeding > 3 months ago	27 (55.1%)	306 (64.3%)	
** Previous admissions**	31 (63.3%)	226 (47.5%)	0.035
Number of previous admissions	2 [2–4]	2 [1–3]	0.05
** History of HIV testing**	23 (46.9%)	93 (19.5%)	< 0.001
**Patient HIV status before enrollment**			
Confirmed HIV positive	16 (32.6%)	0 (0.0%)	< 0.001
HIV exposed not confirmed	9 (18.4%)	8 (1.7%)	
Unknown	24 (49.0%)	468 (98.3%)	

### Prevalence and characteristics of participants with and without HIV infection

The prevalence of HIV infection among admitted children was 9.3% (49 participants), among them 33 (67.3%) were newly diagnosed during the study time. There was a significant age distribution difference between HIV infected and uninfected children with a median age among HIV-infected children being 23 months [IQR 9-27] and 29 months [IQR 15-60] among HIV-uninfected children. Among HIV infected children, those aged 1-24 months constituted a larger proportion 65.3%, followed by those aged 25-59 months (24.5%) and those aged ≥ 60months (10.2%). Distribution of other characteristics is as shown in Table 1 and Table 2.

**Table 2:** Clinical Characteristics of 525 children aged 1 month to 12 years

Characteristics	HIV-infected Number (%) or Median [IQR] *N* = 49	HIV-uninfected Number: (%): or Median [IQR] *N* = 476	*P*-Value
**Presenting symptoms and signs**			
Fever	36 (73.5%)	333 (70.0%)	0.609
Cough	26 (53.1%)	173 (36.3%)	0.022
Difficulty in breathing	14 (28.6%)	110 (23.1%)	0.391
Weight loss	25 (51.0%)	119 (25.0%)	< 0.001
Lethargy	3 (6.1%)	28 (5.9%)	0.946
Diarrhea	17 (34.7%)	127 (26.7%)	0.231
Oral thrush	30 (61.2%)	17 (3.6%)	< 0.001
Lymphadenopathy	8 (16.3%)	13 (2.7%)	< 0.001
Hepatomegaly	8 (16.3%)	42 (8.8%)	0.088
Splenomegaly	5 (10.2%)	35 (7.4%)	0.474
**Vital signs**			
Temperature (^O^C)	37 [36.7–37.8]	37 [36.5–38.0]	0.98
Pulse rate	120 [102–149]	119 [102–134]	0.282
Respiratory rate	36 [30–56]	34[26–44]	0.028
**Acute Malnutrition**			
None	6 (12.2%)	219 (46%)	< 0.001
Mild	0 (0.0%)	79 (16.6%)	
Moderate	7 (14.3%)	74 (15.6%)	
Severe	36 (73.5%)	104 (21.8%)	
**Stunting**			
None	7 (15.6%)	170 (45.6%)	< 0.001
Mild	7 (15.6%)	119 (31.9%)	
Moderate	9 (20.0%)	53 (14.2%)	
Severe	22 (48.9%)	31 (8.3%)	

“Table 3” summaries HIV diagnostic test results, WHO HIV/AIDS clinical stage and CD4 values of HIV infected children.

**Table 3:** HIV diagnostic tests, WHO HIV/AIDS clinical stage and CD4 count of 49 HIV-infected children aged 1 month to 12 years

Variable	Number (%)
**HIV diagnostic test results**	
Positive HIV determine test (*N* = 509)	43 (8.4%)
Positive HIV Unigold test (*N* = 43)	16 (37.2%)
Positive HIV DNA PCR (*N* = 43)	17 (39.5%)
**WHO HIV/AIDS clinical stage (*N* = 49)**	
Stage III	10 (20.4%)
Stage IV	39 (79.6%)
**Low CD4 count (*N* = 49)**	41 (83.7%)

### Factors associated with HIV infection

At time of enrolment clinical features that were associated with HIV infection on multivariable analysis were; history of cough (OR 2.40 [1.08-5.31], *p* = 0.031), oral thrush (OR 20.06[8.29-48.52], *p* <0.001), generalized lymphadenopathy (OR 5.61 [1.06-29.56], *p* = 0.042), severe acute malnutrition (OR 6.78 [2.28-20.12], *p* = 0.001), severe stunting (OR 9.09[2.80-29.53], *p* = 0.034) and a having one or both parents deceased (OR 3.62 [1.10-11.87], *p* = 0.034) (Table 4).

**Table 4:** Factors associated with HIV infection among 525 Children aged 2 months to 12 years

Variable Number: (%): or Median [IQR]	HIV- infected (*n* = 49)	HIV- uninfected (*n* = 476)		OR [95% CI] (Univariable)	*p*-value	OR[95% CI] (Multivariable)	*p*-value
**Male gender**	30 (61.2%)	286	(60.1%)	1.05 [0.57–1.92]	0.88		
**Age in categories**							
1–24 months	32 (65.3%)	186 (39.1%)		2.93 [1.58–5.44]	**0.001**	1.66 [0.64–4.27]	0.294
25–59 months	12 (24.5%)	170 (35.7%)		0.58 [0.30–1.15]	0.119		
> 60 months	5 (10.2%)	120 (25.2%)		0.34 [0.13–0.87]	**0.025**	0.75 [0.19–2.97]	0.683
**Place of residence**							
Other than Mwanza	8 (16.3%)	113 (23.7%)		1	1		
Mwanza	41 (83.7%)	363 (76.3%)		1.60 [0.73–3.50]	0.24		
**Caregiver's level of education**							
> 7 years in school	7 (14.3%)	82 (17.2%)		1	1		
≤ 7 years in school	42 (85.7%)	394 (82.8%)		1.25 [0.54–2.88]	0.602		
**Vital status of parents**							
Parents are alive	40(81.6%)	447 (93.9%)		1	1		
One or both parents are deceased	9 (18.4%)	29 (6.1%)		3.47 [1.54–7.83]	**0.003**	3.62 [1.10–11.87]	**0.034**
**HIV-infected mothers who received HAARTduring pregnancy**	6/14 (42.9%)	14 /16(87.5%)		0.11 [0.02–0.66]	0.016*		
**Previous admission**	31 (63.3%)	226 (47.5%)		1.91 [1.04–3.50]	**0.038**	1.38 [0.60–3.13]	0.446
Number of previous Admissions	2 [2–4]	2 [1–3]		1.15 [0.93–1.42]	0.19		
**Breastfeeding** (in last 3 months)	21 (42.9%)	165 (34.7%)		1.41 [0.78–2.57]	0.26		
**Presenting symptoms and signs**							
Fever	36 (73.5%)	333 (70.0%)		1.194 [0.61–2.31]	0.609		
Cough	26 (53.1%)	173 (36.3%)		1.98 [1.10–3.58]	**0.024**	2.40 [1.08–5.31]	**0.031**
Difficulty in breathing	14 (28.6%)	110 (23.1%)		1.33 [0.69–2.56]	0.39		
Weight loss	25 (51.0%)	119 (25.0%)		3.13 [1.72–5.68]	** < 0.001**	0.65 [0.26–1.60]	0.346
Lethargy	3 (6.1%)	28 (5.9%)		1.04 [0.31–3.57]	0.95		
Diarrhea	17 (34.7%)	127 (26.7%)		1.46 [0.78–2.72]	0.23		
Oral thrush	30 (61.2%)	17 (3.6%)		42.6 [20.1–90.4]	** < 0.001**	20.06[8.29–48.52]	** < 0.001**
Lymphadenopathy	8 (16.3%)	13 (2.7%)		6.95 [2.72–17.73]	** < 0.001**	5.61 [1.06–29.56]	**0.042**
Hepatomegaly	8 (16.3%)	42 (8.8%)		2.02 [0.89–4.58]	0.09		
Splenomegaly	5 (10.2%)	35 (7.4%)		1.43 [0.53–3.84]	0.48		
**Acute Malnutrition**							
None	6 (12.2%)	219 (46%)		1	1		
Mild^a^	0 (0.0%)	79 (16.6%)		(-)	(-)		
Moderate	7 (14.3%)	74 (15.6%)		3.45 [1.12–10.60]	**0.03**	2.97 [0.80–10.99]	0.102
Severe	36 (73.5%)	104 (21.8%)		12.6 [5.16–30.93]	** < 0.001**	6.78 [2.28–20.12]	**0.001**
**Stunting**							
None	7 (15.6%)	170 (45.6%)		1	1		
Mild	7 (15.6%)	119 (31.9%)		1.43 [0.49–4.18]	0.56		
Moderate	9 (20.0%)	53 (14.2%)		4.12 [1.47–11.61]	**0.007**	2.90 [0.82–10.17]	0.097
Severe	22 (48.9%)	31 (8.3%)		17.24 [6.78–43.80]	** < 0.001**	9.09 [2.80–29.53]	** < 0.001**

^**a**^Multivariate analysis was not done since the variable was not applicable to all participants

### Three months mortality

The overall proportion of mortality of the admitted children during the study period was 16.2% (85/525). Fifty- four children died during the hospital stay and 31 children died within the three months after discharge. Among the HIV-infected patients, the total number of deaths were 19/49 (38.8%) versus 66/476 (13.9%) in HIV-uninfected patients. Of the fourteen HIV-infected children who died during the hospital stay, 9 (64.3%) had acute gastroenteritis, 3 (21.4%) had Pneumocytis Jirovecii Pneumonia (PJP) and Pulmonary TB, 1 (7.1%) had septic shock, and 1 (7.1%) had sickle cell disease with severe anemia. Among the five HIV-infected children who died within three months after discharge, 2 (30.0%) had discharge diagnosis of malnutrition and 3 (60.0%) had discharge diagnosis of pulmonary TB.

### Linkage to HIV care among HIV-infected patients

Linkage to HIV care was considered as attendance to HIV clinic at least once after the discharge. Out of 49 HIV-infected children, 71.4% (35/49) of them were discharged from the hospital. Twenty five (71.4%) of the 35 discharged HIV-infected children reported to have attended HIV clinic at least once after discharge and among them 52% (13/25) were newly diagnosed with HIV. Median numbers of visits were 2 [IOR 2-4] . Six (17.1%) of the 35 discharged HIV infected children were loss to follow up. Twenty one (60.0%) of 35 discharged HIV patients reported to be taking ART at time of follow up. Of the discharged HIV-infected children aged 2 years and below, 52.4% (11/21) reported to be taking antiretroviral therapy. Reasons for not attending HIV clinic after discharge were also enquired among 4 patients (11.4%) who were reachable at the time of follow up. For one patient, parents were not aware about attending HIV clinic, for another one was due to lack of disclosure among parents and two participants were unable to travel due to sickness.

## Discussion

### Prevalence and factors associated with HIV infection

In this study that included 525 admitted children, the prevalence of HIV infection was evaluated after providing PITC to every child as recommended by the Tanzanian Ministry of Health and Social Welfare. The prevalence of HIV infection among children admitted at Bugando Medical Centre during the study period was 9.3%. This prevalence is lower compared to 19.2% [5], 12.2% [6], 13%[7] that were documented among hospitalized children from studies done in 1996 in Dar-es-Salaam, 2008 in Kilimanjaro and 2012 in Mwanza, respectively. The difference could be explained by the gradual decline of HIV infection rates from 2003 to 2013 among adults as documented in Global Aids Response Country Progress Report of 2014, with stabilization of HIV prevalence in the last two recent surveys depicting a noticeable discrepancy between and within regions of Tanzania [11].

Furthermore in this study HIV infection was prevalent (65.0%) among children aged 24 months and below. Documentation that, more than 50% of HIV-infected children are below 24 months, have also been observed in other studies done in sub-Saharan Africa. A study in done in South Africa reported 58.0% of HIV-infected children were below 24 months [13] and a study done in Nigeria reported 54.4% of HIV-infected children aged less than 24 months [14]. This could probably be explained by the natural history of HIV infection among perinataly infected children whereby 50-60% of them develop symptoms early in life followed by deterioration to AIDS and death by age of 3-5 years [15].

This study has also found that 87.5% (14/16) of HIV negative children were born to HIV positive mothers who received HAART during pregnancy. . Almost similar findings were reported in Kenya that up to 90% of children were born HIV negative following the administration of HAART in mothers [16]. This finding stresses more on the importance and benefit of measures that are in place to help reduce mother to child HIV transmission. Furthermore, among the HIV-infected patients 33 (67.3%) were newly diagnosed during the study time. Almost similar findings were obtained in Soweto, South Africa by Dramowski A et al, where more than 50% of the patients were diagnosed during admission [17]. This finding emphasizes more on the importance of offering PITC services to children with unknown HIV status and those still at risk of acquiring HIV infection through breastfeeding whenever they attend health care facilities so as to reduce missed opportunities.

In this study it was also found that, all (100%) of the HIV-infected children presented with advanced HIV disease i.e. WHO HIV/AIDS stage III and stage IV .This finding is almost similar to that obtained in South Africa where by 93% of HIV-infected children presented with advanced disease [17]. A study done in Nigeria showed a rather lower percentage (75%) of HIV-infected children presenting with advanced HIV disease [18]. The finding in our study that all HIV-infected children presented with advanced disease could be because the study was done among inpatients where very sick patients are seen, but yet it may also imply that HIV infected children are missed to be diagnosed as early as possible and this could be argued with the fact that more than half of the HIV-infected children were newly diagnosed at time of enrollment. When analyzing for factors associated with HIV infection in this study it was found that, HIV-infected children were more likely to have lost a parent or both, present with cough, oral thrush, lymphadenopathy, acute malnutrition and stunting. These results are in line with those in previous studies describing the clinical presentation of HIV infection in children [5, 13, 14, 18–22]. Hence, the presence of these associated factors should heighten the need to screen for HIV infection among children in settings where routine PITC services are not offered.

### In hospital and Post-hospitalization outcomes

The overall three months mortality among HIV-infected children was higher compared in HIV-uninfected patients (38.7% versus 13.9%). The trend of higher mortality rate among HIV-infected children in comparison to their counterparts is also reflected in other studies done in different parts of Africa [13, 17, 18]. However, the percentages of mortality among HIV-infected children in the study were slightly higher when compared to those documented in cited studies. This may be explained by the different methodology used in the study, whereby total number of deaths that occurred during the study period (i.e. inpatient deaths and deaths within three months after discharge) was analysed.

In this study we also assessed for linkage to care, where by at least one visit to specialized HIV care and treatment clinic was regarded as linkage to care. From a total of 35 HIV-infected patients who were discharged alive from the hospital, 71.4% (25/35) of them reported to have attended HIV clinic at least once after discharge, 11.4% (4/35) did not attend HIV clinic after discharge and 17.1% (6/35) of these patients were loss to follow up. This is in contrary to the study done in Zambia which demonstrated a good percentage (99%) of HIV-infected patients that were enrolled into HIV care after PITC (23). The higher percent seen in a study done in Zambia could probably be explained by methodology used where by the nurse counselors were directly involved in referring or even escorting the patients to onsite services including HIV care. Also afterwards there was community follow up that was done by the peer counselors.

However a considerably high percentage (nearly 30%) of children who never attended HIV clinic three months after discharge and those who were lost to follow up needs an in-depth analysis. We would suggest a provision of adherence and counseling services at BMC after identifying eligible HIV-infected children who require antiretroviral therapy together with establishing a follow up system to keep track of HIV-infected children especially those who are newly diagnosed to find out if they are linked to HIV care, via phone call or physical outreach. Moreover, a large longitudinal study is recommended to asses factors that may be associated with poor linkage to HIV care among HIV-infected children post diagnosis.

## Conclusion

Late diagnosis is a reality among children in most settings in sub-Saharan Africa. Providing HIV testing and counseling to every admitted child with unknown HIV status has shown to improve diagnosis, although most children present already in advanced disease. Effective follow up strategies are needed to ascertain that HIV infected children diagnosed from inpatient hospital settings are correctly linked to specialized HIV care and treatment clinics, as many are lost to follow up only 3 months after discharge and another 40% is not on ART despite the severity of the disease.

### Definition of terms

Acute gastroenteritis is defined as the inflammation of the mucus membranes of the gastrointestinal tract and is characterized by diarrhea or vomiting of less than 14 days.

Pneumocystis Jirovecii Pneumonia (PJP), formerly referred to as Pneumocystis Carinii Pneumonia (PCP) is a serious fungal infection of the lungs that most commonly affects the immunocompromised individuals such as those with HIV infection

Pulmonary tuberculosis is a bacterial infection due to Mycobacterium tuberculosis, spread from person to person through inhalation of infected respiratory droplets.

Septic shock is defined as sepsis associated with hypotension and perfusion abnormalities despite the provision of adequate fluid (volume) resuscitation

Sickle cell disease is an inherited blood disorder that affects shape the red blood cells into sickled cells.

Acute malnutrition is a rapid onset condition characterized by sudden weight loss or bilateral pitting edema due to either inadequate energy or protein intake.

Stunting is the impaired growth and development that children experience from poor nutrition, repeated infection, and inadequate psychosocial stimulation. Children are defined as stunted if their height-for-age is more than two standard deviations below the WHO Child Growth Standards median.

## Data Availability

All data generated or analysed during this study are included in this published article [and its supplementary information files].

## Abbreviations

AIDS: Acquired Immunodeficiency Syndrome
ART: Antiretroviral Therapy
BMC: Bugando Medical Centre
CD4: Cluster of differentiation 4
CTC: Care and Treatment Clinics
DBS: Dried Blood Spot
DNA PCR: Deoxyribonucleic acid polymerase chain reaction
ELISA: Enzyme Linked Immunosorbent Assay
HAART: Highly Active Antiretroviral Therapy
HIV: Human Immunodeficiency Virus
IQR: Interquartile Ranges
PITC: Provider Initiated Testing and Counselling
PMTCT: Prevention of Maternal to Child Transmission
SSA: Sub-Saharan Africa
WHO: World Health Organization
